# Effect of apoA-I Mutations in the Capacity of Reconstituted HDL to Promote ABCG1-Mediated Cholesterol Efflux

**DOI:** 10.1371/journal.pone.0067993

**Published:** 2013-06-27

**Authors:** Georgios Daniil, Vassilis I. Zannis, Angeliki Chroni

**Affiliations:** 1 Institute of Biosciences and Applications, National Center for Scientific Research “Demokritos”, Agia Paraskevi, Athens, Greece; 2 Molecular Genetics, Departments of Medicine and Biochemistry, Whitaker Cardiovascular Institute, Boston University School of Medicine, Boston, Massachusetts, United States of America; Harvard Medical School, United States of America

## Abstract

ATP binding cassette transporter G1 (ABCG1) mediates the cholesterol transport from cells to high-density lipoprotein (HDL), but the role of apolipoprotein A-I (apoA-I), the main protein constituent of HDL, in this process is not clear. To address this, we measured cholesterol efflux from HEK293 cells or J774 mouse macrophages overexpressing ABCG1 using as acceptors reconstituted HDL (rHDL) containing wild-type or various mutant apoA-I forms. It was found that ABCG1-mediated cholesterol efflux was severely reduced (by 89%) when using rHDL containing the carboxyl-terminal deletion mutant apoA-I[Δ(185–243)]. ABCG1-mediated cholesterol efflux was not affected or moderately decreased by rHDL containing amino-terminal deletion mutants and several mid-region deletion or point apoA-I mutants, and was restored to 69–99% of control by double deletion mutants apoA-I[Δ(1–41)Δ(185–243)] and apoA-I[Δ(1–59)Δ(185–243)]. These findings suggest that the central helices alone of apoA-I associated to rHDL can promote ABCG1-mediated cholesterol efflux. Further analysis showed that rHDL containing the carboxyl-terminal deletion mutant apoA-I[Δ(185–243)] only slightly reduced (by 22%) the ABCG1-mediated efflux of 7-ketocholesterol, indicating that depending on the sterol type, structural changes in rHDL-associated apoA-I affect differently the ABCG1-mediated efflux of cholesterol and 7-ketocholesterol. Overall, our findings demonstrate that rHDL-associated apoA-I structural changes affect the capacity of rHDL to accept cellular cholesterol by an ABCG1-mediated process. The structure-function relationship seen here between rHDL-associated apoA-I mutants and ABCG1-mediated cholesterol efflux closely resembles that seen before in lipid-free apoA-I mutants and ABCA1-dependent cholesterol efflux, suggesting that both processes depend on the same structural determinants of apoA-I.

## Introduction

Apolipoprotein A-I (apoA-I) is the major protein component of high-density lipoprotein (HDL) [1]. ApoA-I contains 22- and 11-amino acid repeats which based on x-ray crystallography are organized in amphipathic α-helices [2], [3]. ApoA-I plays an essential role in the biogenesis, structure, function, and plasma concentration of HDL [1]. HDL assembles by an initial ATP binding cassette transporter A1 (ABCA1) mediated transfer of cellular phospholipids and cholesterol to extracellular lipid-poor apoA-I acceptor. The initial lipidation of apoA-I is followed by remodeling in the plasma compartment of HDL particles by the esterification of cholesterol by the enzyme lecithin: cholesterol acyltransferase, exchange between HDL and other lipoproteins of apolipoproteins and lipids as well as putative transfer of additional cellular cholesterol to the growing particles by the scavenger receptor class B type I (SR-BI) and the cell surface transporter ATP binding cassette transporter G1 (ABCG1) (reviewed in [1]).

Overexpression of ABCG1 has been shown to promote efflux of cellular cholesterol to HDL particles [4], [5]. Additional studies in *abcg1* deficient mice also suggested that ABCG1 plays a critical role in the efflux of cellular cholesterol to HDL [5]. Moreover, it was determined that ABCG1 played a critical role in promoting macrophage reverse cholesterol transport *in vivo*, assessed by intraperitoneal injection of mice with [^3^H]cholesterol-labeled J774 macrophages with either increased or reduced ABCG1 expression, as well as primary macrophages lacking ABCG1 expression, and measurement of the macrophage-derived [^3^H]cholesterol levels in plasma and feces [6]. However, it was shown that plasma lipid and lipoprotein levels were not affected in *abcg1* deficient and *ABCG1* transgenic mice, indicating that although ABCG1 mediates cholesterol efflux, it may not affect plasma levels of HDL [5], [7]. Nonetheless, a recent study using high-density genotyping arrays containing single-nucleotide polymorphisms suggested an association between HDL-cholesterol levels in humans and ABCG1 [8]. The animal studies suggested that loss of *αbcg1* in mice results in massive lipid accumulation in hepatocytes and in macrophages within multiple tissues, with the more pronounced effect observed in pulmonary macrophages [5], [7], [9]–[11]. In addition, ABCG1 was shown to promote efflux of 7-ketocholesterol and related oxysterols from macrophages and endothelial cells to HDL, protecting cells against dysfunction and apoptosis [12], [13]. Recent genetic association studies in humans identified functional variants in ABCG1 associated with increased risk of coronary artery disease [14], supporting an important role of ABCG1 in atherosclerosis development and cardiovascular disease. Furthermore, HDL from cholesteryl ester transfer protein (CETP) deficient subjects or patients treated with the CETP inhibitors torcetrapib or anacetrapib was shown to have enhanced ability to promote ABCG1-mediated cholesterol efflux from macrophages [15], [16], indicating that ABCG1 may participate in the atheroprotective properties of HDL including cellular sterol efflux capacity.

To date, the role of apoA-I, the main protein constituent of HDL, in the ability of HDL to serve as acceptor for ABCG1-mediated sterol efflux remains unclear. The purpose of the present study was to study whether specific domains in apoA-I are involved in ABCG1-mediated cholesterol and oxysterol efflux and in this way to gain insight into the ABCG1-mediated sterol efflux mechanism. To this end, we analyzed the ability of reconstituted HDL (rHDL) containing various recombinant apoA-I forms lacking amino-terminal, carboxyl-terminal or internal domains, as well as carrying point mutations to promote ABCG1-mediated efflux of cellular cholesterol and 7-ketocholesterol. Our findings suggest that although the central helices of apoA-I bound to rHDL are sufficient to promote ABCG1-mediated cholesterol efflux, ABCG1-mediated efflux of cholesterol and to a much lesser effect of 7-ketocholesterol is decreased by deletion of the carboxyl-terminal domain from full-length apoA-I bound to rHDL. Measurement of plasma membrane micro-fluidity of ABCG1-expressing cells showed differences between cells labeled with cholesterol and 7-ketocholesterol, indicating that ABCG1 may differentially affect the distribution of cholesterol and 7-ketocholesterol within plasma membrane, where they become accessible for removal by rHDL containing apoA-I. Furthermore, our studies showed that all apoA-I mutants tested in lipid-free form or as components of rHDL display similar pattern in their capacity to promote ABCA1- and ABCG1-mediated cholesterol efflux, respectively, suggesting that the two efflux processes may share common steps.

## Materials and Methods

### Materials

Fetal bovine serum (FBS), plasmin, 1-palmitoyl-2-oleoyl-L-phosphatidylcholine (POPC), sphingomyelin, cholesterol, 5-cholesten-3β-ol-7-one (7-ketocholestrol) and phenylmethylsulfonyl fluoride (PMSF) were purchased from Sigma-Aldrich Corporation (St. Louis, MO, USA). Dulbecco’s modified Eagle’s medium (DMEM) was from Lonza Biowhitaker (Verviers, Belgium). Opti-MEM I medium, Lipofectamine-2000 and 1-pyrenedodecanoic acid were from Life Technologies (Carlsbad, CA, USA). *Not*I and *Kpn*I were from Fermentas (St. Leon-Rot, Germany). Quick T4 DNA ligase was from New England Biolabs (Beverly, MA, USA). 4-[^14^C]Cholesterol (0.1 mCi/ml, specific activity 50 mCi/mmol), 1,2,6-[^3^H]7-ketocholesterol (1 mCi/ml, specific activity 40 Ci/mmol) were obtained from ARC (St. Louis, MO, USA). 5-aminoimidazole-4-carboxyamide ribonucleoside (AICAR) was from Cayman Chemicals (Ann Arbor, MI, USA). Mouse monoclonal anti-human apoA-I antibody 5F6 was from University of Ottawa Heart Institute, Canada and goat anti-mouse IgG coupled to HRP was from Novagen, San Diego, CA, USA. All other reagents were purchased from Sigma- Aldrich Corporation, Invitrogen, Bio-Rad (Hercules, CA, USA), Fisher Scientific (Schwerte, Germany) or other standard commercial sources.

### Production and Purification of Recombinant apoA-I and Preparation of rHDL Containing POPC/cholesterol/apoA-I or POPC/sphingomyelin/cholesterol/apoA-I

All plasmids, recombinant baculoviruses and recombinant adenoviruses containing the wild-type (WT) and mutated human apoA-I genes were constructed as described previously [17]–[22]. ApoA-I was purified from baculovirus-infected Sf-9 cells (Life Technologies) or the culture medium of adenovirus-infected HTB-13 cells (ATCC, Manassas, VA, USA) as described previously [17]–[22].

Reconstituted discoidal HDL (rHDL) particles were prepared by the sodium cholate dialysis method [23] using POPC/cholesterol/apoA-I/sodium cholate in a molar ratio of 100∶10∶1∶100 or 80∶10∶1∶100 and POPC/sphingomyelin/cholesterol/apoA-I/sodium cholate in a molar ratio of 100∶10∶10∶1∶100, as previously described [24]. Apolipoprotein-lipid complex formation was verified by analysis with 4–20% native polyacrylamide gradient gel electrophoresis at 4°C.

### ABCG1-expressing Plasmid Construction

The plasmid expressing the human ABCG1 transporter was generated by subcloning human full-length ABCG1 cDNA from pCMV-sport6 vector (IMAGE:5198758, Imagenes, Berlin, Germany) into the corresponding sites of pcDNA 3.1(+) (Invitrogen). The DNA fragment was digested with *Not*I and *Kpn*I and ligated with quick T4 DNA ligase. The cDNA sequence was confirmed by DNA sequencing.

### Cholesterol and 7-ketocholesterol Efflux Assays in HEK293 Cells Transfected with an ABCG1 Expressing Plasmid

Human embryonic kidney (HEK) 293 cells (ATCC) were cultured in DMEM (ultra-glutamine 1 and 4.5 g/L glucose) supplemented with 10% (v/v) FBS and antibiotics. HEK293 cells were plated on 24-well plates coated with poly-D-lysine at a density of 1×10^5^ cells/well. The next day, cells at about 95% confluence were transfected with the pcDNA3.1(+) plasmid encoding human ABCG1 or pcDNA3.1(+) plasmid (mock) using Lipofectamine 2000, according to the manufacturer’s instructions. The DNA-Lipofectamine 2000 complexes were formed in serum-free Opti-MEM I. Four to five hours after DNA-Lipofectamine 2000 addition the medium was removed and the cells were labeled with 0.5 ml labeling medium (0.3 µCi/ml 4-[^14^C]cholesterol or 1.6 µCi/ml 1,2,6-[^3^H]7-ketocholesterol in DMEM, without antibiotics, supplemented with 10% (v/v) heat inactivated FBS) and incubated for 24 h. At the end of this period, the cells were washed twice with PBS and incubated for 1 h with DMEM supplemented with 0.2% (w/v) fatty acid-free BSA. Subsequently, the cells were incubated with 0.4 ml of DMEM supplemented with 0.2% (w/v) BSA with or without various concentrations of rHDL containing WT or mutant apoA-I forms at 37°C for 4 h. In some experiments the cells were cholesterol loaded using 30 µg/ml acetylated LDL (acLDL) (Molecular Probes/Life Technologies) added in the labeling medium. The rest of the procedure was same as described above.

For cholesterol efflux experiments, the media were collected and clarified by centrifugation in a microcentrifuge for 5 min, and the radioactivity in 300 µl of the supernatant was determined by liquid scintillation counting. The cells were lysed with 1 ml 0.1 M NaOH, and the radioactivity of the total amount of the cell lysate was determined by scintillation counting. [^14^C]Cholesterol efflux was expressed as the percentage of the radioactivity released in the medium relative to the total radioactivity in cells and medium. To calculate the net ABCG1-mediated cholesterol efflux, the cholesterol efflux of the cells transfected with the control plasmid (mock) was subtracted from the cholesterol efflux of the cells transfected with the ABCG1-expressing plasmid.

For 7-ketocholesterol efflux experiments, at the end of the 4 h incubation period in the presence or absence of rHDL, the media were collected and clarified by centrifugation in a microcentrifuge for 5 min. A 100 µL aliquot of the supernatant was first extracted with chloroform and methanol [25] and then, the radioactivity of the chloroform phase was determined by liquid scintillation counting. The cells were lysed by 800 µL of lysis buffer (PBS containing 0.1% (v/v) Triton X-100) for 30 min at room temperature. Radioactivity was measured in 100 µL of lysate, following extraction [25]. [^3^H]7-Ketocholesterol efflux was expressed as the percentage of the radioactivity released in the medium relative to the total radioactivity in cells and medium. To calculate the net ABCG1-mediated 7-ketocholesterol efflux, the 7-ketocholesterol efflux of the cells transfected with the control plasmid (mock) was subtracted from the 7-ketocholesterol efflux of the cells transfected with the ABCG1-expressing plasmid.

### Cholesterol and 7-ketocholesterol Efflux Assays in J774 Macrophages

J774 mouse macrophages (ATCC) were cultured in DMEM (ultra-glutamine 1 and 4.5 g/L glucose) supplemented with 10% (v/v) FBS and antibiotics. J774 macrophages were plated in 24-well plates at density 2.5×10^5^ cells/well. The following day, cells were labeled with 0.5 ml labeling medium (0.3 µCi/ml 4-[^14^C]cholesterol or 1.6 µCi/ml 1,2,6-[^3^H]7-ketocholesterol in DMEM supplemented with 0.2% (w/v) BSA). Following 24 h of labeling, the cells were washed twice with serum-free medium and equilibrated for 24 h with or without 1 mM 5-aminoimidazole-4-carboxyamide ribonucleoside (AICAR) [26] in 0.5 ml of DMEM supplemented with 0.2% (w/v) BSA. At the end of the treatment period with AICAR, the cells were washed twice with serum-free medium and incubated with 0.5 ml of DMEM, supplemented with 0.2% (w/v) BSA, with or without 1 µΜ rHDL containing WT or mutant apoA-I forms at 37°C for 4h. In some experiments the cells were cholesterol loaded using 30 µg/ml acLDL (Molecular Probes/Life Technologies) added in the labeling medium. The rest of the procedure was same as described above.

For cholesterol efflux experiments, the media were collected and clarified by centrifugation in a microcentrifuge for 5 min. The radioactivity in 55 µl of the supernatant was determined by liquid scintillation counting. Cells were lysed in 400 µl of lysis buffer (PBS containing 1% (v/v) Triton X-100) for 30 min at room temperature and radioactivity of the total amount of cell lysate was determined by liquid scintillation counting. [^14^C]cholesterol efflux was expressed as the percentage of the radioactivity released in the medium relative to the total radioactivity in cells and medium. To calculate the net ABCG1-mediated cholesterol efflux, the cholesterol efflux of the untreated cells was subtracted from the cholesterol efflux of the cells treated with AICAR.

For 7-ketocholesterol efflux experiments, at the end of the 4 h incubation period in the presence or absence of rHDL, the media were collected and clarified by centrifugation in a microcentrifuge for 5 min. A 100 µL aliquot of the supernatant was first extracted with chloroform and methanol [25] and then, the radioactivity of the chloroform phase was determined by liquid scintillation counting. The cells were lysed by 800 µL of lysis buffer (PBS containing 0.1% (v/v) Triton X-100) for 30 min at room temperature. Radioactivity was measured in 100 µL of lysate, following extraction [25]. [^3^H]7-Ketocholesterol efflux was expressed as the percentage of the radioactivity released in the medium relative to the total radioactivity in cells and medium. To calculate the net ABCG1-mediated 7-ketocholesterol efflux, the 7-ketocholesterol efflux of the untreated cells was subtracted from the 7-ketocholesterol efflux of the cells treated with AICAR.

### Detection of ABCG1

Cells (HEK293 or J774) were lysed in lysis buffer (50 mM Tris-HCl, pH 7.5, 150 mM NaCl, 1% Nonidet P-40, 0.25% deoxycholate and protease inhibitors). Cell lysates, normalized for cell protein content, were subjected to 10% SDS-PAGE, and the resolved proteins were transferred to nitrocellulose membrane for Western blotting. ABCG1 was detected using the rabbit anti-ABCG1 polyclonal antibody (NB400–132, Novus Biologicals, Cambridge, UK) and a goat anti-rabbit IgG coupled to horseradish peroxidase (GE Healthcare, Pittsburgh, PA, USA). To normalize the ABCG1 western blot signal, blots were re-blotted with mouse anti β-tubulin monoclonal antibody TUB 2.1 (Sigma) followed by incubation with goat anti-mouse IgG-horseradish peroxidase (Novagen/Millipore, Darmstadt, Germany). Densitometric analysis was performed using the Image J image analysis software (NIH, Bethesda, MD, USA) [27].

### Proteolysis of rHDL

The proteolysis of rHDL containing the WT apoA-I carried out as described previously [28] with some modifications. rHDL (∼1.2 mg protein/ml) was incubated with 0.008 U/ml of human plasmin in 150 mM NaCl, 1 mM EDTA, 5 mM Tris, pH 7.4 at 37°C for 1 h. Proteolysis was stopped by adding PMSF at a final concentration of 2 mM. Aliquots of the incubation mixtures were analyzed by 15% SDS-PAGE. ApoA-I was detected by Coomassie Blue staining or Western blot analysis using the mouse monoclonal anti-human apoA-I antibody 5F6 (epitope 118–141 aa) and a goat anti-mouse IgG coupled to HRP. The proteolysed rHDL was used in cholesterol efflux experiments in HEK293 cells transfected with an ABCG1-expressing plasmid.

### 1-Pyrenedodecanoic Acid Fluorescence Measurements

1-Pyrenedodecanoic acid labeling of HEK293 cells plasma membranes and fluorescence measurements were carried out as described [29]–[31]. HEK293 cells were transfected with an ABCG1-expressing plasmid or a mock plasmid and incubated with cholesterol or 7-ketocholestrol at the same concentrations and conditions as described above for the sterol efflux experiments, except that non-radioactive sterols were used. At the end of the incubation, cells were washed twice and suspended in PBS. 1-pyrenedodecanoic acid was added to a fluorimeter cuvette containing cells suspension to a final concentration of 2 µM and incubated for 5 min in the dark. Τhe fluorescence intensity was scanned, in a Hitachi Science and Technology F2500 Fluorescence spectrometer, from 380–580 nm at an excitation wavelength of 340 nm, using a 2.5 nm excitation slit width and 2.5 nm emission slit width. After scanning, the ratio of the maximum fluorescent intensities of excimer to pyrene monomer was calculated at 475 and 397 nm, respectively.

### Statistical Analysis

All results are reported as mean±SD. Parameters were compared between two groups using unpaired, two-tailed *t*-test. p values are indicated in the figure legends.

## Results

### rHDL Capacity to Promote ABCG1-mediated Cholesterol Efflux from HEK293 Cells Transfected with an ABCG1-expressing Plasmid: Effects of Amino-terminal, Carboxyl-terminal or Internal Deletion Mutants as well as Point Mutants of apoA-I

**Figure 1:**
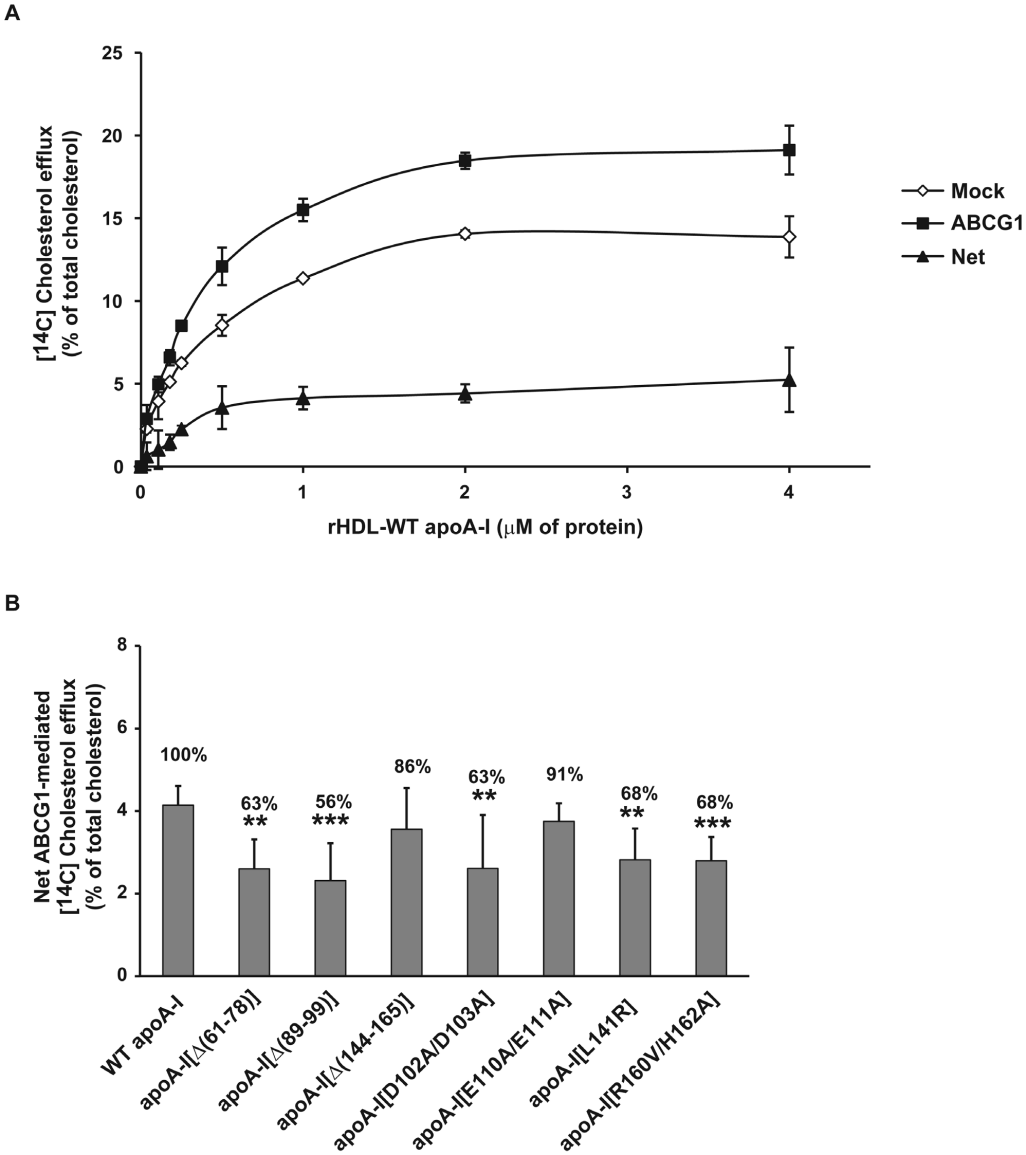
Effect of internal deletion or point mutations of apoA-I bound to rHDL particles on ABCG1-mediated cholesterol efflux from HEK293 cells transfected with an ABCG1-expressing plasmid. (A, B) HEK293 cells were transfected with empty vector (mock) or with ABCG1-expressing plasmid, labeled with [^14^C]cholesterol for 24 h and then incubated with rHDL containing WT or mutants apoA-I forms, at various concentrations (A) or 1 µM of apoA-I (B), for 4 h. The net ABCG1-mediated [^14^C]cholesterol efflux is calculated as the difference in percent of cholesterol efflux between ABCG1-transfected and mock-transfected cells. The numbers on top of the bars represent the net ABCG1-mediated cholesterol efflux relative to the WT control set to 100% (B). Values are the means ± SD from six independent experiments performed in duplicate. **, p < 0.01 vs WT apoA-I; ***, p < 0.001 vs WT apoA-I.

Several previous studies have investigated the role of lipid-free or lipid-associated apoA-I domains in ABCA1- or SR-BI-dependent cholesterol efflux processes, respectively [18], [19], [21], [32], [33]. However, nothing is known about the involvement of apoA-I in ABCG1-mediated cholesterol efflux by HDL. To determine whether specific domains of apoA-I affect the capacity of rHDL to promote ABCG1-mediated cholesterol efflux we prepared rHDL particles containing the WT or various mutant apoA-I forms, phospholipid and cholesterol at initial POPC:cholesterol (C):apoA-I molar ratio of 100∶10∶1, as described previously [18]. A variety of apoA-I mutants lacking the amino-terminal, carboxyl- terminal, both amino- and carboxyl-terminal or internal domains, as well as carrying point mutants in the central helices were used in these studies. These rHDL preparations were incubated with HEK293 cells transfected with an empty vector (mock) or an ABCG1-expressing plasmid and labeled with [^14^C]-cholesterol. The net ABCG1-mediated cholesterol efflux was obtained by subtracting the efflux values of the mock-transfected HEK293 cells from the ABCG1-transfected cells. Monitoring of cholesterol efflux using rHDL containing the WT apoA-I at various protein concentrations showed that net ABCG1-mediated cholesterol efflux reaches a plateau above concentrations of 0.5 µM protein (Figure 1A). The percent of net ABCG1-mediated cholesterol efflux values for rHDL containing WT apoA-I (at a concentration of 1 µM apoA-I) was found similar to that described previously [4], [34]. To facilitate the analysis of the cholesterol efflux by rHDL containing the different apoA-I forms the net ABCG1-mediated cholesterol efflux values obtained for WT apoA-I was set to 100%. As shown in Figure 1B, rHDL (at a saturating concentration of 1 µM apoA-I) containing several mid-region deletion and point apoA-I mutants (apoA-I[Δ(61–78)], apoA-I[Δ(89–99)], apoA-I[Δ(144–165)], apoA-I[D102A/D103A], apoA-I[E110A/E111A], apoA-I[L141R] and apoA-I[R160V/H162A]) moderately decreased (by 9–44%) the ABCG1-mediated cholesterol efflux as compared to rHDL containing WT apoA-I.

**Figure 2:**
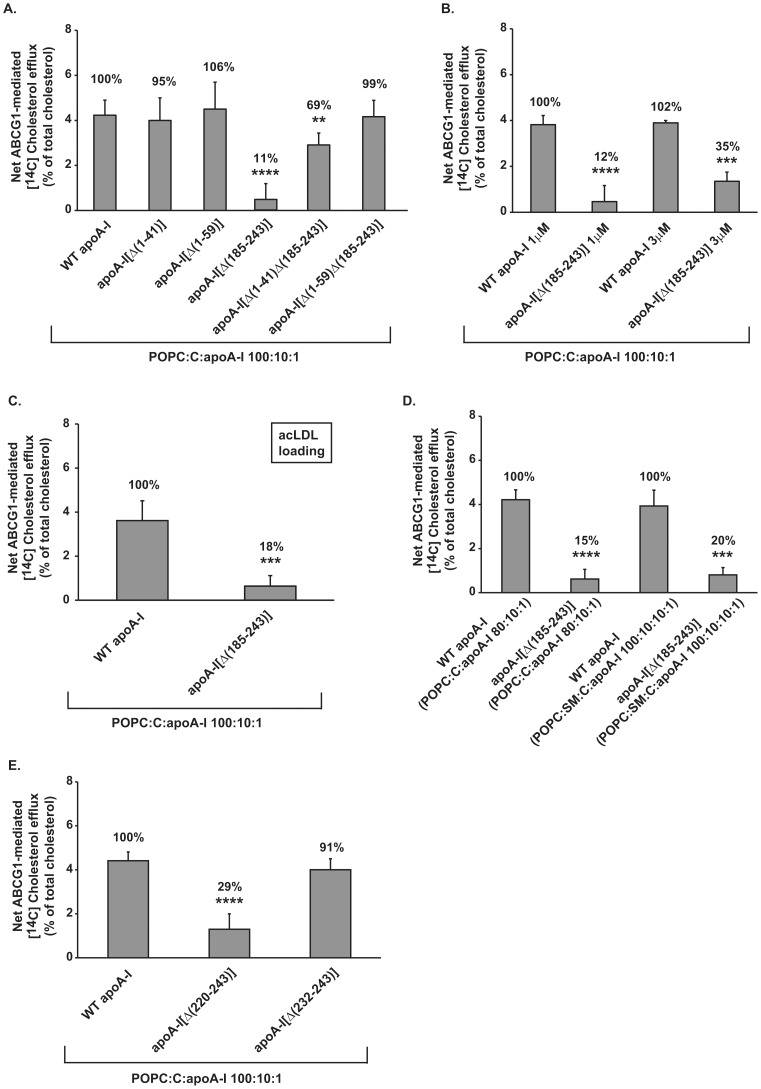
Effect of amino-, carboxyl-terminal deletions or double deletions of both the amino- and carboxy-terminal regions of apoA-I bound to rHDL particles on ABCG1-mediated cholesterol efflux from HEK293 cells transfected with an ABCG1-expressing plasmid. HEK293 cells were transfected with empty vector (mock) or with ABCG1-expressing plasmid, labeled with [^14^C]cholesterol (A, B, D, E) or [^14^C]cholesterol and 30 µg/ml acLDL (C) for 24 h and then incubated with rHDL containing WT or mutants apoA-I forms, at a concentration of 1 µM apoA-I (A-E) or 3 µM apoA-I (B), for 4 h. rHDL particles have various lipid (POPC, SM, C):apoA-I ratios as indicated in each panel. The net ABCG1-mediated [^14^C]cholesterol efflux is calculated as described in Figure 1. (A) Values are the means ± SD from six (A, E) or three (B) independent experiments performed in duplicate or two (C, D) independent experiments performed in triplicate. **, p < 0.01 vs WT apoA-I; ***, p < 0.001 vs WT apoA-I; ****, p ≤ 0.0001 vs WT apoA-I. POPC, 1-palmitoyl-2-oleoyl-L-phosphatidylcholine; C, cholesterol; SM, sphingomyelin.

Analysis of the effect of rHDL containing the carboxyl-terminal deletion mutant apoA-I[Δ(185–243)] showed that the ABCG1-mediated cholesterol efflux was decreased by 89% in the presence of 1 µM protein of rHDL containing this apoA-I mutant (Figure 2A). To compensate for any difference in particle size or composition, ABCG1-mediated cholesterol efflux was also measured in the presence of apoA-I[Δ(185–243)]-containing rHDL at a much higher concentration (3 µM) than the expected saturating concentration. As shown in Figure 2B ABCG1-mediated cholesterol efflux is still impaired (decrease by 65%) in the presence of carboxyl-terminal deletion apoA-I mutant. Further analysis, using cells loaded with acLDL also showed that apoA-I[Δ(185–243)]-containing rHDL (at a concentration of 1 µM apoA-I) has greatly reduced (by 82%) capacity to promote ABCG1-mediated cholesterol efflux (Figure 2C). Measurement of the lipid and protein composition of rHDL particles showed that the rHDL particles containing WT apoA-I or apoA-I[Δ(185–243)] have similar final molar ratio of POPC:C:apoA-I (86∶9∶1 for WT apoA-I and 91∶10∶1 for apoA-I[Δ(185–243)]) and their sizes vary slightly as determined by nondenaturing polyacrylamide gel electrophoresis (populations of ∼12.2 nm and ∼10 nm for WT apoA-I, ∼12.2 nm, ∼11 nm and ∼9 nm for apoA-I[Δ(185–243)]). It has been shown previously that at a given phospholipid or protein concentration rHDL particles with sizes ranging from 7.8 to 17.0 nm are similarly efficient in promoting cholesterol efflux from ABCG1 overexpressing cells [35]. In addition, other studies have demonstrated that net ABCG1-mediated cholesterol efflux was either unchanged regardless of the phospholipid: protein ratio [36] or that it increased upon increasing phospholipid content of the acceptor [37]. Therefore, based on the composition and size of the apoA-I[Δ(185–243)]-containing rHDL it would be expected that these particles display either similar or even increased ABCG1-mediated cholesterol efflux capacity compared to WT apoA-I-containing rHDL. Thus, our opposite finding of severely reduced cholesterol efflux induced by this mutant suggests that it lacks structural features that are necessary to endow rHDL with the capacity to accept cholesterol from cells overexpressing ABCG1.

To study further whether the lipid content of rHDL particles containing the apoA-I[Δ(185–243)] mutant may affect the ABCG1-mediated cholesterol efflux we prepared rHDL containing POPC, C and apoA-I at an initial molar ratio of 80∶10∶1 or POPC, sphingomyelin (SM), C and apoA-I at an initial molar ratio of 100∶10∶10∶1. Measurement of the lipid and protein composition showed that the rHDL particles containing WT or mutant apoA-I forms have similar final molar ratio of phospholipid:C:apoA-I. This ratio is 72∶8∶1 and 72∶9∶1, respectively, for WT apoA-I or apoA-I[Δ(185–243)]-containing rHDL that were made with POPC, C, apoA-I and 99∶8∶1 and 97∶9∶1, respectively, for WT apoA-I or apoA-I[Δ(185–243)]-containing rHDL that were made with POPC+SM, C, apoA-I. As shown in Figure 2D the ABCG1-mediated cholesterol efflux in the presence of 1 µM protein of rHDL containing the apoA-I[Δ(185–243)] mutant, prepared at initial molar ratio of POPC:C:apoA-I 80∶10∶1, was greatly reduced (by 85%) similarly to the results obtained by rHDL containing the apoA-I[Δ(185–243)] mutant prepared at an initial molar ratio of POPC:C:apoA-I 100∶10∶1. This is in agreement with previous studies which showed that all rHDL particles containing POPC:apoA-I at various ratios of 40–178∶1 are similarly efficient in promoting cholesterol efflux from ABCG1 overexpressing cells [35]. In addition, the ABCG1-mediated cholesterol efflux is also impaired (decrease by 80%) in the presence of 1 µM protein of rHDL containing the apoA-I[Δ(185–243)] prepared at an initial molar ratio of POPC:SM:C:apoA-I 100∶10∶10∶1 (Figure 2D). Overall, our findings suggest that all apoA-I[Δ(185–243)]-containing rHDL studied here, which were prepared with various phospholipid content or lipid:protein ratio, display greatly reduced (by 80–89%) capacity to promote ABCG1-mediated cholesterol efflux compared to WT apoA-I-containing rHDL.

For a more detailed localization of the domain within the carboxyl-terminal region of apoA-I, that affects the capacity of rHDL-associated apoA-I to promote ABCG1-mediated cholesterol efflux, we studied the capacity of rHDL-associated apoA-I[Δ(220–243)] and apoA-I[Δ(232–243)] (at a concentration of 1 µM apoA-I) to promote ABCG1-mediated cholesterol efflux. The ABCG1-mediated cholesterol efflux was 29% of the WT control value for the mutant lacking the 220–243 domain and restored to 91% of the WT control value for the mutant lacking the 232–243 domain (Figure 2E). These data suggest that amino acid residues 220–231 of apoA-I are possibly of the most important for the ability of the full-length apoA-I as a component of rHDL to promote ABCG1-mediated cholesterol efflux.

Analysis of the effect of rHDL (at a concentration of 1 µM apoA-I) containing amino-terminal deletion mutants apoA-I[Δ(1–41)] or apoA-I[Δ(1–59)] showed that the ABCG1-mediated cholesterol efflux was not affected, while cholesterol efflux was restored to 69–99% of the WT apoA-I control value by double deletion mutants apoA-I[Δ(1–41)Δ(185–243)] and apoA-I[Δ(1–59)Δ(185–243)] (Figure 2A). This finding suggests that the central helices of apoA-I can be sufficient to form rHDL particles that can promote ABCG1-mediated cholesterol efflux. Overall, our findings suggest a structure-function relationship for rHDL-associated apoA-I mutants and ABCG1-mediated cholesterol efflux.

### Effect of the Carboxyl-terminal Deletion Mutant apoA-I[Δ(185–243)] on ABCG1-mediated Cholesterol Efflux from J774 Mouse Macrophages Following Treatment with AICAR

**Figure 3:**
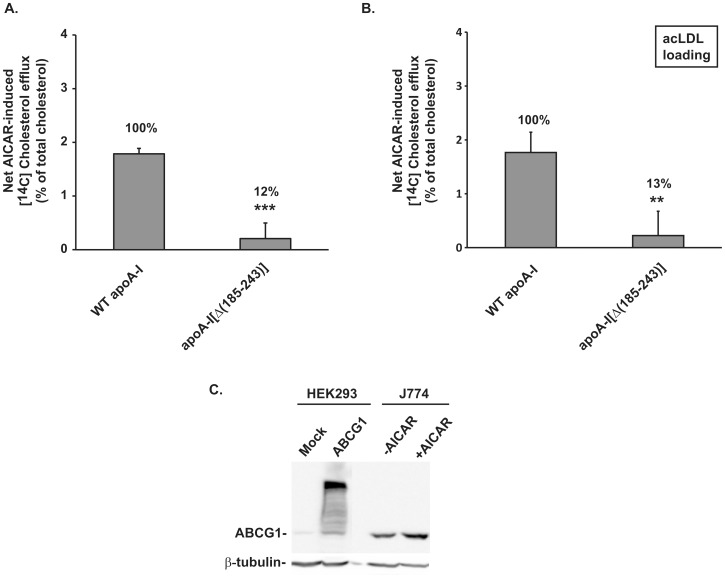
Effect of the carboxyl-terminal deletion mutant apoA-I[Δ(185–243)] bound to rHDL particles on ABCG1-mediated cholesterol efflux from J774 mouse macrophages following treatment with AICAR. J774 mouse macrophages were labeled with [^14^C]cholesterol (A) or [^14^C]cholesterol and 30 µg/ml acLDL (B) for 24 h and then incubated with 1 mM AICAR for 24 h. At the end of this incubation period the cells were incubated with rHDL containing WT apoA-I or apoA-I[Δ(185–243)], at a concentration of 1 µM apoA-I, for 4 h. The net AICAR-induced (ABCG1-mediated) [^14^C]cholesterol efflux is calculated as the difference in percent of cholesterol efflux between untreated and AICAR-treated cells. Values are the means ± SD from three independent experiments performed in duplicate. **, p < 0.01 vs WT apoA-I; ***, p < 0.001 vs WT apoA-I. (C) Western blot analysis, performed as described in “Materials and Methods”, showing the increase in ABCG1 protein levels after AICAR treatment of J774 macrophages. ABCG1 protein levels of HEK293 cells transfected with empty vector (mock) or with ABCG1-expressing plasmid are also shown for comparison.

To confirm the effect of the carboxyl-terminal deletion mutant apoA-I[Δ(185–243)] on ABCG1-mediated cholesterol efflux on another, more physiologically relevant, cell type we measured cholesterol efflux from J774 mouse macrophages that had been labeled with [^14^C]-cholesterol and treated with or without 5-aminoimidazole-4-carboxyamide ribonucleoside (AICAR). AICAR has been reported to significantly increase ABCG1 mRNA and protein levels without affecting mRNA and protein expression of ABCA1, scavenger receptors including scavenger receptor-A (SRA), CD36, SR-BI and cholesterol synthesis related genes in J774 macrophages [26]. AICAR treatment of J774 macrophages caused a two-fold increase in ABCG1 protein levels (Figure 3C), as it has been reported previously [26]. As shown in Figure 3A, similar to the results obtained in HEK293 cells, apoA-I[Δ(185–243)]-containing rHDL decreased (by 88%) the AICAR-induced (ABCG1-mediated) cholesterol efflux from J774 mouse macrophages. Furthermore, we measured ABCG1-mediated cholesterol efflux from acLDL loaded J774 macrophages, in which cholesterol may be derived from different cellular pools as compared to J774 macrophages simply labeled with [^14^C]-cholesterol [38]. As shown in Figure 3B, ABCG1-mediated cholesterol efflux is still impaired (decrease by 87%) in the presence of apoA-I[Δ(185–243)]-containing rHDL. Overall, our findings suggest that full-length apoA-I as a component of rHDL requires its carboxyl-terminal domain in order to promote efflux of cellular cholesterol by an ABCG1-mediated process.

### Effect of the Carboxyl-terminal Deletion Mutant apoA-I[Δ(185–243)] on ABCG1-mediated Efflux of Cellular 7-ketocholesterol from HEK293 Cells Transfected with an ABCG1-expressing Plasmid and from J774 Mouse Macrophages Following Treatment with AICAR

**Figure 4:**
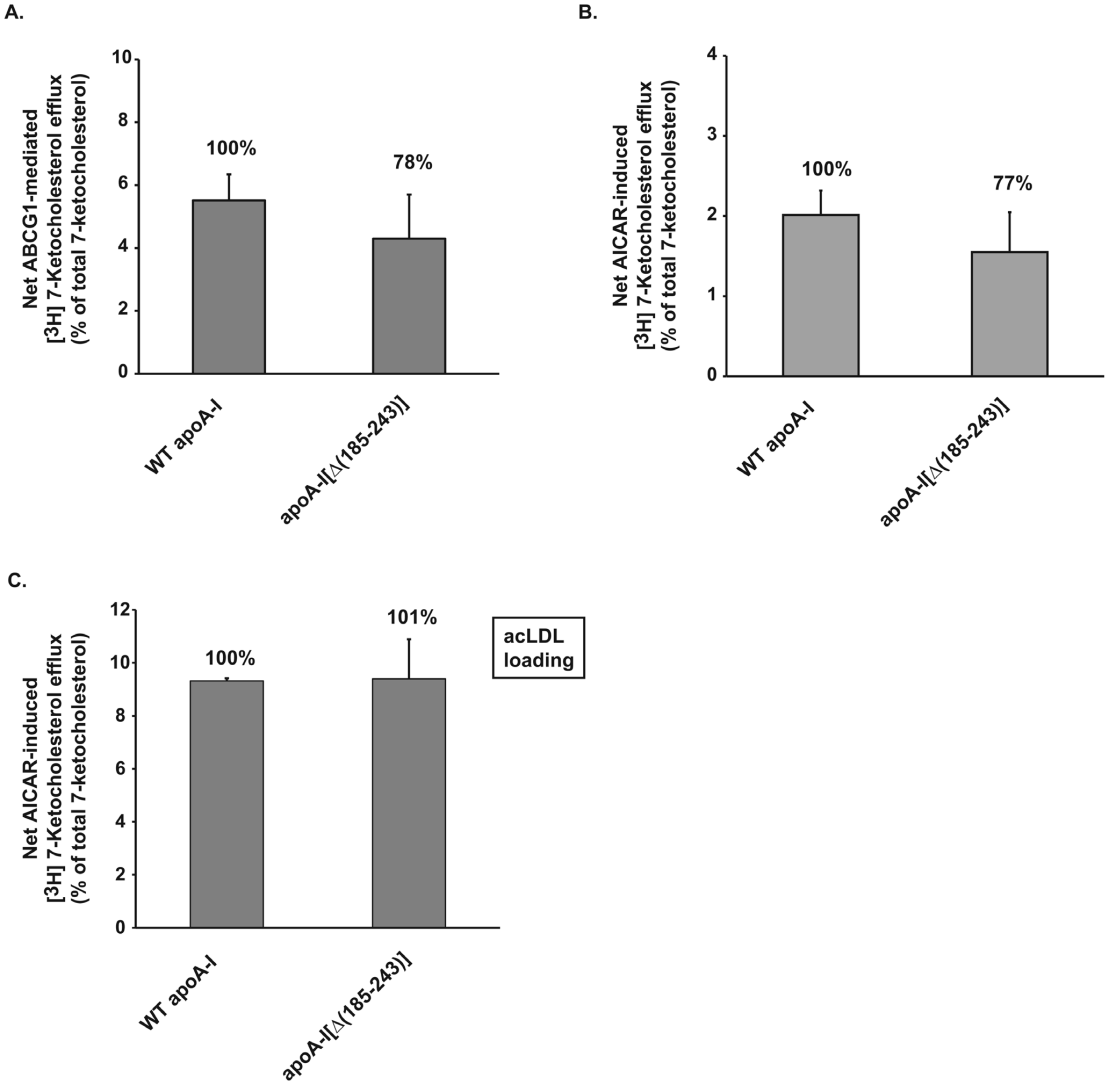
Effect of the carboxyl-terminal deletion mutant apoA-I[Δ(185–243)] bound to rHDL particles on ABCG1-mediated 7-ketocholesterol efflux from HEK293 cells transfected with an ABCG1-expressing plasmid and from J774 mouse macrophages following treatment with AICAR. (A) HEK293 cells were transfected with empty vector (mock) or with ABCG1 plasmid, labeled with [^3^H]7-ketocholesterol for 24 h and then incubated with rHDL containing WT apoA-I or apoA-I[Δ(185–243)], at a concentration of 1 µM apoA-I, for 4 h. (B, C) J774 mouse macrophages were labeled with [^3^H]7-ketocholesterol (B) or [^3^H]7-ketocholesterol and 30 µg/ml acLDL (C) for 24 h and then incubated with 1 mM AICAR for 24 h. At the end of this incubation period the cells were incubated with rHDL containing WT apoA-I or apoA-I[Δ(185–243)], at a concentration of 1 µM apoA-I, for 4 h. The net ABCG1-mediated [^3^H]7-ketocholesterol efflux is calculated as the difference in percent of 7-ketocholesterol efflux between ABCG1-transfected and mock-transfected cells (A) or between untreated and AICAR-treated cells (B, C). Values are the means ± SD from three independent experiments performed in duplicate.

In addition to cholesterol efflux, ABCG1 has been shown to promote cellular efflux of 7-ketocholesterol and related oxysterols [12], [13]. To explore whether carboxyl-terminal deletion mutant apoA-I[Δ(185–243)] affects cholesterol and 7-ketocholesterol efflux to a similar degree, we performed 7-ketocholesterol efflux studies in mock- or ABCG1-transfected HEK293 cells using apoA-I[Δ(185–243)]-containing rHDL as 7-ketocholesterol acceptor. The analysis showed that, in contrast to its effect on cholesterol efflux, apoA-I[Δ(185–243)]-containing rHDL only moderately decreased (by 22%) the ABCG1-mediated 7-ketocholesterol efflux (Figure 4A). Similar to the results obtained in HEK293 cells, apoA-I[Δ(185–243)]-containing rHDL also moderately decreased (by 23%) or not affected the AICAR-induced (ABCG1-mediated) 7-ketocholesterol efflux from J774 mouse macrophages without or with acLDL preloading, respectively (Figures 4B, C). Our findings indicate that, depending on the sterol type, structural changes in rHDL-associated apoA-I affect differently the ABCG1-mediated efflux of cholesterol and 7-ketocholesterol.

### Effect of Cholesterol and 7-ketocholesterol on Cellular Membrane Fluidity of ABCG1-expressing HEK293 Cells Probed by 1-pyrenedodecanoic Acid

**Figure 5:**
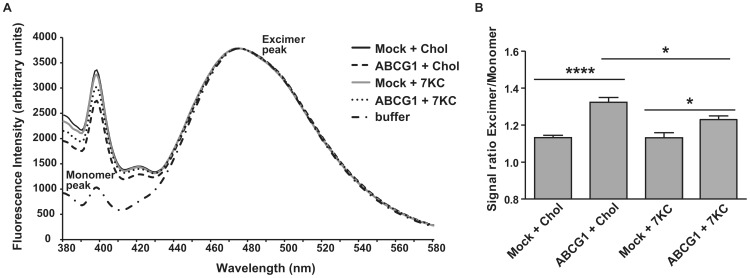
Plasma membrane fluidity, probed by 1-pyrenedodecanoic acid, of HEK293 cells labeled with cholesterol or 7-ketocholesterol. HEK293 cells were transfected with empty vector (mock) or with ABCG1-expressing plasmid, incubated with cholesterol or 7-ketocholestrol at the same concentrations and conditions used for the sterol efflux experiments and then suspended in PBS and mixed with the probe 1-pyrenedodecanoic acid as described in “Materials and Methods”. (A) Fluorescence emission spectra of 1-pyrenedodecanoic acid in the presence or absence of HEK293 cells. The fluorescence spectrum of the probe exhibits two major peaks, one at 475 nm and one at 397 nm, corresponding to the excimer and monomer state of the probe. The monomer peak is low when the probe is in aqueous buffer, but is significantly enhanced when the probe is added onto cell suspension. Characteristic spectra of 6–9 separate experiments are shown. (B) The ratio of excimer to monomer peak was measured in the presence of mock or ABCG1-transfected HEK293 cells labeled with cholesterol or 7-ketocholestrol. Values are the means ± SD of 6–9 separate experiments. Chol: cholesterol; 7 KC: 7-ketocholesterol. *, p<0.05; ****, p<0.0001.

In order to gain insight into the protein-membrane interactions that allow apoA-I[Δ(185–243)]-containing rHDL to remove 7-ketocholesterol, but not cholesterol, from cells expressing ABCG1 we measured plasma membrane microfuidity. The lateral and transversal fluidity of the cellular membrane can be altered by changes in the lipid composition of the membrane. 1-Pyrenedodecanoic acid excimers break into monomers in the lipophilic interior of the membrane which give distinct fluorescence spectra and this property has been used to gain insights on the micro-fluidic state of the membrane [29], [30]. To obtain insight on the micro-fluidic properties of the cellular membrane of mock- or ABCG1-transfected HEK293 cells when labeled with cholesterol or 7-ketocholesterol we measured the fluorescence of 1-pyrenedodecanoic acid added onto the cell suspension. We found no change in the excimer/monomer ratio of the probe between mock-transfected HEK293 cells that had been labeled with cholesterol or 7-ketocholesterol. In contrast, we found a statistically significant increase in the excimer/monomer ratio of 1-pyrenedodecanoic acid of 17% between mock- and ABCG1-transfected cells labeled with cholesterol and of 8% between mock- and ABCG1-transfected cells labeled with 7-ketocholesterol (Figure 5). These findings suggest different changes in the lateral and transversal fluidity of the cellular membrane between ABCG1-transfected HEK293 cells labeled with cholesterol and 7-ketocholesterol. These differences may be related to different plasma membrane distribution between cholesterol and 7-ketocholesterol in ABCG1-expressing cells that may affect the sterol accessibility and the sterol acceptor capacity of apoA-I[Δ(185–243)]-containing rHDL.

### Effect of apoA-I Proteolysis on the Ability of rHDL Particles to Promote ABCG1-mediated Cholesterol Efflux

**Figure 6:**
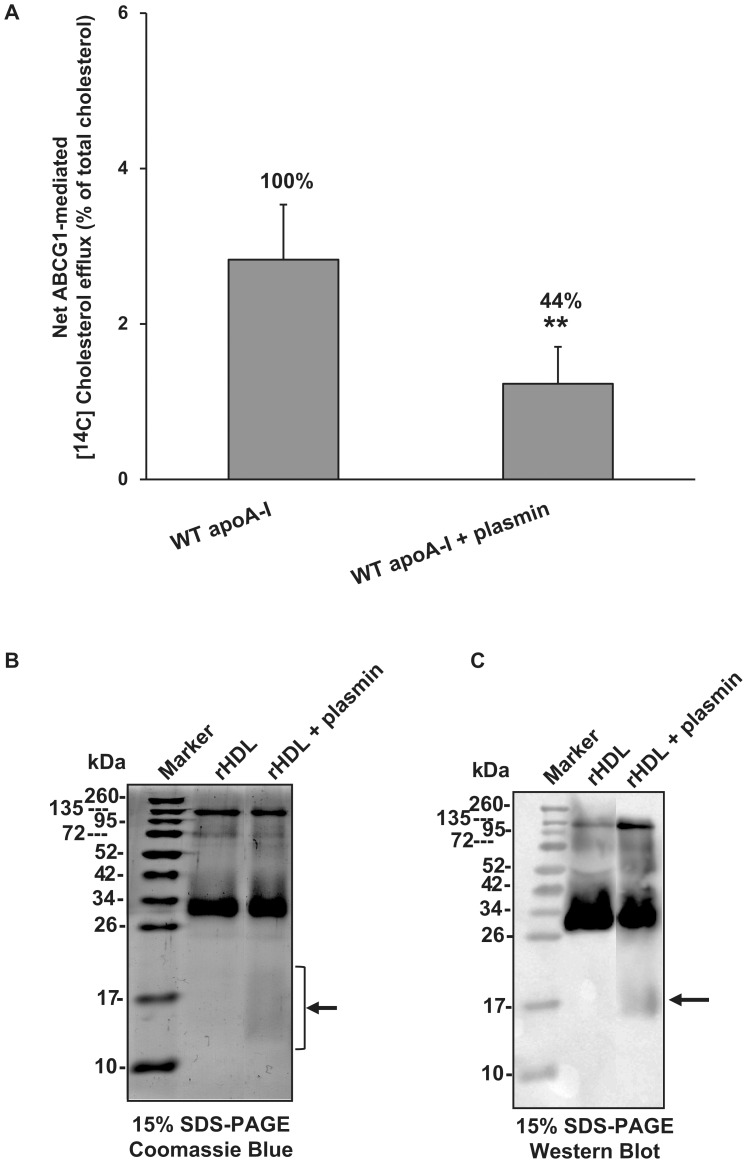
Effect of plasmin on the ability of rHDL particles containing WT apoA-I to promote ABCG1-mediated cholesterol efflux. (A) HEK293 cells were transfected with empty vector (mock) or with ABCG1-expressing plasmid, labeled with [^14^C]cholesterol for 24 h and then incubated with 1 µM of untreated rHDL-associated apoA-I or rHDL-associated apoA-I that had been treated with 0.008 U/ml of human plasmin at 37°C for 1 h as described in “Materials and Methods”. After incubation of the cells with the rHDL preparations for 4 h the net ABCG1-mediated [^14^C]cholesterol efflux is calculated as described in Figure 1. Values are the means ± SD from two independent experiments performed in triplicate. **, p < 0.01 vs WT apoA-I. (B, C) Aliquots of plasmin-treated rHDL were analyzed by 15% SDS–PAGE. ApoA-I was detected by Coomassie Blue staining (B) or Western blot analysis (C) as described in “Materials and Methods”. The arrows indicate apoA-I fragments.

Previous studies showed that several proteases (matrix metalloproteinases, plasmin, kallikrein, and mast cell chymase and tryptase) known to be present in the human arterial intima can degrade the apoA-I of HDL_3_ in vitro, thereby impairing HDL-mediated cholesterol efflux from macrophage foam cells [28], [39]–[41]. In addition, it was shown that proteolysis of lipidated apoA-I by plasmin and some metalloproteinases generated apoA-I fragments lacking the carboxyl-terminal region [40], [42]. Thus, we examined whether the proteolytic degradation of apoA-I in rHDL can impair the ABCG1-mediated cholesterol efflux by rHDL. For this purpose, we incubated 1 µM rHDL-associated apoA-I with 0.008 U/ml of human plasmin at 37°C for 1 h and then used this rHDL preparation to measure cholesterol efflux from mock- and ABCG1-transfected HEK293 cells (Figure 6A). Plasmin at a concentration of 0.008 U/ml caused mild proteolysis as documented by slight loss of rHDL protein and faint bands of about 14–21 kDa (Figure 6B, C), also described previously [28], [42]. Smaller concentration of plasmin (0.004 U/ml) had no apparent effect on apoA-I, while higher concentration (0.04 U/ml) degraded apoA-I almost completely. Treatment of rHDL with plasmin resulted in a significant decrease (by 56% as compared to untreated rHDL) of ABCG1-mediated cholesterol efflux (Figure 6A). This decrease in rHDL cholesterol efflux capacity could not be attributed to the observed small decrease in WT apoA-I levels, since the rHDL concentration used in cholesterol efflux assay is above the saturating levels (Figure 1A), but most possibly to the presence of truncated apoA-I forms that lead to structural changes in rHDL-associated apoA-I that could affect the ABCG1-mediated efflux of cholesterol. This finding suggests that a potential proteolysis of the major protein of HDL by arterial intima proteases may impair the capacity of HDL to promote ABCG1-mediated cellular cholesterol.

## Discussion

HDL has been shown to induce efflux of cholesterol from cellular plasma membranes by an ABCG1-mediated process [4], [5]. The purpose of the current study was to identify critical domains or residues of apoA-I which may affect the capacity of rHDL to promote ABCG1-depedent sterol efflux and thus to gain further insight on the mechanisms by which ABCG1 promotes the removal of cholesterol or oxysterols from cells. We have generated a large number of apoA-I mutants that can be assigned in four main categories: (i) amino- or carboxyl-terminal deletions; (ii) double deletions of both the amino- and the carboxyl-terminal regions; (iii) internal deletions in helices H1, H2, H3 and H6 (helix designation is described on [3]); and (iv) point mutations in helices H4, H5 and H6. With such extensive mutagenesis, one might expect to identify whether there are critical domains or residues of apoA-I, which are involved in ABCG1-mediated cholesterol efflux. Furthermore, these apoA-I mutants in lipid-free form have been previously studied for their capacity to promote ABCA1-dependent cholesterol efflux [19], [21], [22]. Thus, the use of these mutants in the current study allows us to examine whether the cholesterol transporters ABCA1 and ABCG1 share the same or different specificity for domains in apoA-I that are required for cholesterol efflux.

Our analysis showed that in ABCG1-transfected HEK293 cells or AICAR-stimulated J774 macrophages, cholesterol efflux was strongly impaired by rHDL particles containing the carboxyl-terminal deletion mutant apoA-I[Δ(185–243)]. Previous studies indicated that there is little or no specificity for the acceptor of cholesterol since LDL, HDL_2_, HDL_3_, phospholipid/apoA-I particles of various sizes and small unilamellar particles can function as acceptors for cholesterol from cells in an ABCG1-mediated manner [35], [36]. However, our data show for the first time that apoA-I in rHDL is involved in the process of ABCG1-mediated cholesterol efflux. Analysis of rHDL composition and size suggests that the impairment of ABCG1-mediated cholesterol efflux is not due to major differences in particle composition or size between rHDL particles containing WT apoA-I or apoA-I[Δ(185–243)]. Therefore, our findings indicate that structural changes in discoidal rHDL-associated apoA-I could reduce the ABCG1-mediated cholesterol efflux capacity of rHDL. Future studies using spherical HDL particles containing the various apoA-I mutant forms, prepared *in vitro* or *in vivo*, will further enhance our understanding of the relationship between structural changes of HDL-associated apoA-I and ABCG1-mediated cholesterol efflux.

The ABCG1-mediated cholesterol efflux was not affected by rHDL containing amino-terminal deletion mutants (apoA-I[Δ(1–41)] and apoA-I[Δ(1–59)]) and moderately decreased by rHDL containing several mid-region deletion or point apoA-I mutants. Surprisingly however, the ABCG1-mediated cholesterol efflux defect of rHDL-associated apoA-I was restored to 69–99% of the WT apoA-I control value when, in addition to the carboxyl-terminus, the amino-terminus of apoA-I was also deleted. Physicochemical analysis of rHDL particles containing the apoA-I[Δ(1–41)Δ(185–243)] mutant have shown that the α-helical content of this double deletion mutant is 78% in rHDL particles, whereas the α-helical content of the WT apoA-I is 67% in rHDL particles [19]. In another study similar analysis of POPC/apoA-I particles the α-helical content of the amino-terminal deletion mutant apoA-I[Δ(1–43)] showed a small decrease (helicity 73%), while the α-helical content of the carboxyl-terminal deletion mutant apoA-I[Δ(190–243)] showed a greater decrease (helicity 71%) compared to WT apoA-I (helicity 76%) [43] **.** Hence, it is possible that conformational changes of apoA-I in rHDL affect the capacity of rHDL to promote ABCG1-mediated cholesterol efflux.

In a previous study, we found that the ABCA1-dependent cholesterol efflux was greatly reduced by the lipid-free carboxyl-terminal mutants apoA-I[Δ(185–243)] and apoA-I[Δ(220–243)], while it was slightly reduced by the lipid-free amino-terminal deletion mutants apoA-I[Δ(1–41)] and apoA-I[Δ(1–59)] and restored to 75–80% of the WT apoA-I control value by the lipid-free double amino- and carboxyl-terminal deletion mutants apoA-I[Δ(1–41)Δ(185–243)] and apoA-I[Δ(1–59)Δ(185–243)] [19]. Several internal deletions and point mutants of apoA-I (apoA-I[Δ(61–78)], apoA-I[Δ(89–99)], apoA-I[Δ(144–165)], apoA-I[D102A/D103A], apoA-I[E110A/E111A], apoA-I[L141R] and apoA-I[R160V/H162A]) in lipid-free form did not affect or moderately decreased the ABCA1-dependent cholesterol efflux [21], [22]. The current data show that all the apoA-I mutants studied as components of rHDL forms affect the ABCG1-mediated cholesterol efflux in a similar pattern as the same lipid-free apoA-I mutants affect the ABCA1-dependent cholesterol efflux. The finding that all lipid-free apoA-I mutants that promote ABCA1-dependent cholesterol efflux promote also ABCG1-mediated cholesterol efflux as components of rHDL supports previous studies which proposed that lipidation of apoA-I by ABCA1 generates a cholesterol acceptor for ABCG1-mediated cholesterol efflux [37], [44], [45].

Based on the similar effect of apoA-I mutations on ABCA1- and ABCG1-mediated cholesterol efflux one could speculate that ABCA1 and ABCG1 cholesterol transporters may share common steps of the cholesterol efflux process. The mechanism by which ABCG1 promotes sterol efflux to extracellular acceptors has not been resolved. Initial studies had suggested that ABCG1 is localized to both the plasma membrane and internal membrane structures [34], [46], [47], while more recent studies suggested that ABCG1 is localized to endosomes and recycling endosomes [48], [49]. It has been proposed that ABCG1 could transport sterols across the bilayer of endosomes before their fusion with the plasma membrane, resulting in redistribution of these sterols to the outer leaflet of the plasma membrane thus allowing for subsequent efflux of sterols to HDL or other acceptors [34], [49]. In contrast to the proposed intracellular localization of ABCG1, ABCA1 localizes to the plasma membrane [50] where it interacts with apoA-I and promotes efflux of phospholipid and cholesterol [21]. Whether ABCA1-mediated lipid efflux is a pure cell surface event or it involves retroendocytosis and intracellular lipidation remains unclear (reviewed in [51]). It is also unclear whether ABCA1 co-transports phospholipids and cholesterol or mediates only the phospholipid efflux, followed by the efflux of cholesterol in an ABCA1-regulated but ABCA1-independent process [51]. The similar cholesterol efflux capacity of lipid-free and lipidated apoA-I mutants could favor a model of ABCA1-dependent lipid efflux in which lipid-free apoA-I accepts lipids in an ABCA1-mediated process, gets lipidated, changes its structural conformation in the presence of lipids and subsequently accepts more cholesterol from membrane pools generated by ABCA1. Similarly, HDL may accept cholesterol from plasma membrane pools formed by ABCG1.

Besides cholesterol efflux, ABCG1 promotes efflux of 7-ketocholesterol and related oxysterols [12], [13]. The overexpression of ABCG1 in cells is expected to increase the concentration of 7-ketocholesterol in the outer leaflet of the plasma membrane where it becomes accessible to exogenous acceptors [49]. 7-Ketocholesterol contains an additional oxygen molecule compared to cholesterol, which changes the three-dimensional shape of the molecule, increases the polarity, and alters the orientation of the molecule in a membrane [52]. We detected differences in the micro-fluidic properties of the cellular membrane between ABCG1-expressing cells labeled with cholesterol or 7-ketocholesterol. Other studies on the effects of cholesterol and 7-ketocholesterol on membrane properties suggested that compared to cholesterol, 7-ketocholesterol can differentially modulate membrane biophysical properties and thus influence functions involved in protein-membrane interaction [53], [54]. Furthermore, it was shown that 7-ketocholesterol translocates much more rapidly than cholesterol between phospholipid vesicles and this behavior was attributed to weakened association with the membrane bilayer [55]. Our data showed that the carboxyl-terminal deletion mutant apoA-I[Δ(185–243)]-containing rHDL, that has diminished ABCG1-mediated cholesterol efflux capacity, only moderately decreased the ABCG1-mediated 7-ketocholesterol efflux. It is thus suggested that contrary to the ABCG1-mediated cholesterol efflux, ABCG1-mediated 7-ketocholesterol efflux has little or no specificity for the conformation of apoA-I on rHDL.

Based on the different capacity of rHDL-associated apoA-I that lacks its carboxyl-terminal region to promote ABCG1-mediated cholesterol and 7-ketocholesterol efflux one can envision the following steps for ABCG1-mediated sterol efflux: firstly, ABCG1, regardless of its cellular localization, transports sterols to the outer leaflet of the plasma membrane and subsequently, rHDL-associated apoA-I[Δ(185–243)] interacts with the plasma membrane in order to bind and remove 7-ketocholesterol, which is loosely associated with the plasma membrane compared to cholesterol [55]. In contrast, rHDL-associated apoA-I[Δ(185–243)] cannot remove easily the more tightly associated to the plasma membrane cholesterol, which requires as acceptor rHDL-associated apoA-I with a favorable structural conformation. Alternatively, based on the similar pattern in the capacity of rHDL-associated apoA-I mutants to promote ABCG1-mediated cholesterol efflux with that observed for lipid-free apoA-I mutants and ABCA1-mediated cholesterol efflux, one can suggest the localization of ABCG1 in the plasma membrane and interaction with lipoprotein acceptors. However, this localization should be transient, at least in ABCG1 overexpressing cells, since previous studies failed to detect specific HDL-association in BHK or HEK293 cells overexpressing the human ABCG1 [4], [36]. In any case, the observation that rHDL-associated apoA-I that lacks its carboxyl-terminal region has a reduced capacity to promote ABCG1-mediated cholesterol efflux compared to rHDL-associated full-length apoA-I has to be taken into consideration in our efforts to interpret the mechanism of ABCG1-mediated cholesterol efflux.

Proteolysis of HDL-associated apoA-I at the carboxyl-terminal site has been shown to occur by the action of plasmin or various recombinant metalloproteinases *in vitro* or by metalloproteinases secreted from macrophages [40], [42], [56]. The proteolysis of apoA-I by metalloproteinases produced various fragments, including fragments with a mass of approximately 22 kDa that correspond to apoA-I cleaved after residues 191 or 188 [40], [56]. Such proteolysis of HDL-associated apoA-I *in vivo* by proteases (metalloproteinases and plasmin) that are present in the human arterial intima [57], [58] could yield apoA-I fragments similar to the apoA-I[Δ(185–243)] mutant studied here and thus may impair their capacity when remain associated with HDL to promote ABCG1-mediated cholesterol efflux from macrophages. In macrophages, ABCG1, and not SR-BI or ABCA1, has been shown recently to be primarily responsible for free cholesterol mobilization to rHDL [59]. In addition, plasmin and metalloproteinases are also released by alveolar macrophages [60], [61]. Since the lungs of *Abcg1* deficient mice accumulate macrophage foam cells that contain high levels of cholesterol [5], [10], the carboxyl-terminal truncation of HDL-associated apoA-I by alveolar macrophages proteases could diminish the ABCG1-mediated cholesterol efflux process and result in lipid accumulation in lungs.

In summary, our findings suggest that the ABCG1-mediated efflux of cholesterol, but not of 7-ketocholesterol, shows specificity for structural domains of apoA-I bound to rHDL. Specifically, we showed that although the mid region alone of apoA-I associated to rHDL can promote ABCG1-mediated cholesterol efflux, deletion of carboxyl-terminal region 185–243 from full-length apoA-I diminishes ABCG1-mediated cholesterol efflux. This finding may have physiological significance since proteolysis of HDL-associated apoA-I *in vivo* may affect the capacity of HDL to promote cholesterol efflux from macrophages. The exact mechanism underlying the effect of structural changes of HDL-associated apoA-I and the impact of proteolysis of HDL on ABCG1-mediated cholesterol efflux will require further studies.
