# Correction: Effect of apoA-I Mutations in the Capacity of Reconstituted HDL to Promote ABCG1-Mediated Cholesterol Efflux

**DOI:** 10.1371/journal.pone.0108141

**Published:** 2014-09-08

**Authors:** The PLOS ONE Staff

In the Funding statement, there are some missing funders. Please refer to the complete Funding statement below:

Funding for this work was provided by the General Secretariat of Research and Technology of Greece (Synergasia 09SYN-12-897) Synergasia 2009 programme (project code: 09SYN-12-897) which is co-funded by the European Regional Development Fund and Greek national resources, the Ministry of Education, Lifelong Learning and Religious Affairs of Greece (Thalis 3569 MIS 377286), the Hellenic Society of Lipidology, Atherosclerosis and Vascular Disease and the National Institutes of Health (grant HL48739). GD received financial support from the graduate student fellowship program of National Center for Scientific Research ‘‘Demokritos’’. The funders had no role in study design, data collection and analysis, decision to publish, or preparation of the manuscript.
